# Good Adherence to the Mediterranean Diet Lowered Risk of Renal Glomerular Impairment in Children: A Longitudinal Study

**DOI:** 10.3390/nu14163343

**Published:** 2022-08-15

**Authors:** Menglong Li, Huidi Xiao, Wen Shu, Nubiya Amaerjiang, Jiawulan Zunong, Dayong Huang, Yifei Hu

**Affiliations:** 1 Department of Child, Adolescent Health and Maternal Care, School of Public Health, Capital Medical University, Beijing 100069, China; 2 Department of Hematology, Beijing Friendship Hospital, Capital Medical University, Beijing 100050, China

**Keywords:** Mediterranean diet, KIDMED test, kidney impairment, children, China

## Abstract

Healthy diet patterns have a positive effect on chronic non-communicable diseases in the pediatric population, but the evidence is limited on the association between kidney impairment and adherence to a Mediterranean diet. We aim to determine the associations between Mediterranean diet adherence and longitudinal tubular and glomerular impairment in children. Based on four waves of urine assays conducted from October 2018 to November 2019, we assayed urinary β_2_-microglobulin (β_2_-MG) and microalbumin (MA) excretion to determine transient renal tubular and glomerular impairment during the follow-up of the child cohort (PROC) study in Beijing, China. We assessed Mediterranean diet adherence using the 16-item Mediterranean Diet Quality Index in children and adolescents (KIDMED) among 1914 primary school children. Poor, intermediate, and good adherence rates for the Mediterranean diet were 9.0% (KIDMED index 0–3), 54.4% (KIDMED index 4–7) and 36.5% (KIDMED index 8–12), respectively. A short sleep duration was more prevalent in children with lower Mediterranean diet adherence, with no significant differences presenting in the other demographic and lifestyle covariates. The results of linear mixed-effects models showed that a higher urinary MA excretion was inversely associated with a higher KIDMED score (β = −0.216, 95%CI: −0.358, −0.074, *p* = 0.003), after adjusting for sex, age, BMI z-score, SBP z-score, screen time, sleep duration and physical activity. Furthermore, in generalized linear mixed-effects models, consistent results found that transient renal glomerular impairment was less likely to develop in children with intermediate Mediterranean diet adherence (aOR = 0.68, 95%CI: 0.47, 0.99, *p* = 0.044) and in children with good Mediterranean diet adherence (aOR = 0.60, 95%CI: 0.40, 0.90, *p* = 0.014), taking poor Mediterranean diet adherence as a reference. We visualized the longitudinal associations between each item of the KIDMED test and kidney impairment via a forest plot and identified the main protective eating behaviors. Children who adhere well to the Mediterranean diet have a lower risk of transient glomerular impairment, underscoring the necessity of the early childhood development of healthy eating patterns to protect kidney health.

## 1. Introduction

Dietary patterns highlight the habitual consumption of foods, which are associated with the four leading causes of death— coronary heart disease, stroke, some cancers, and type II diabetes—in developed countries and are dramatically increasing in developing countries as well [1,2]. As a modifiable factor, a diet pattern can be categorized as “healthy” or “unhealthy” [1,3,4]. The Mediterranean diet is one of the healthy diet patterns based on a variety of whole foods, including fruits and vegetables, whole grains, legumes, nuts and seeds, and fishes, compared with highly processed foods [3]. Good adherence to the Mediterranean diet has a positive role in protecting people from chronic non-communicable diseases (NCDs) [5,6,7].

Early development and adherence to a healthy eating pattern is critical for children’s growth and kidney health [8,9,10,11]. Western-style diets including fast food or fried high-energy foods have increased dramatically over the past few decades, gaining popularity among the younger generation in China [4]. It leads to a gradual decrease in the adoption of the traditional Chinese dietary pattern, which is similar in composition to the Mediterranean diet [12]. Diet measurement is often challenging, especially in children, given dietary patterns and food diversity and complexity, and the Mediterranean Diet Quality Index in children and adolescents (KIDMED) [13] provides an optimal and standardized tool to assess a healthier food mix. The Mediterranean diet has been strongly advocated in recent years to address the upsurging early onset of NCDs. However, the KIDMED index has not been well-practiced in Chinese children.

Substantial evidence from studies in the pediatric population has shown that adherence to the Mediterranean diet as assessed by the KIDMED index is associated with a reduced risk of obesity [14], metabolic syndrome [15,16], diabetes [17,18] and early cardiovascular diseases [19], while daily lifestyles may have a synergistic effect [20,21]. Paucity of evidence has shown the association between Mediterranean diet adherence and kidney impairment. We found only one cross-sectional study examining the association of Mediterranean diet adherence with the albuminuria level in Greek adolescents [11]. Albuminuria, which is assessed using elevated microalbumin (MA) excretion, reflects early renal glomerular impairment [22], and an elevated β_2_-microglobulin (β_2_-MG) reflects early renal tubular impairment [23]. In this study, we aim to determine the longitudinal associations between transient kidney impairment and Mediterranean diet adherence in children using the KIDMED index.

## 2. Materials and Methods

### 2.1. Study Design and Participants

Based on the child cohort (PROC) study, we enrolled 1914 children aged 6 to 8 years attending primary school in Beijing, China (detailed elsewhere [24]). We conducted 4 waves of urine assays from October 2018 to November 2019 (detailed elsewhere [25,26]). In brief, the first wave of urine assays was performed from October to November 2018 and waves 2–4 were performed within one week in November 2019 (see details elsewhere [27]). All participants provided at least one urine sample, for a sample size of 6968 visits across 4 waves.

### 2.2. Urine Collection and Measurements

The urine collection and measurement procedures are detailed elsewhere [25,26]. Briefly, we performed a baseline fasting urine assay for wave 1, a 24 h urine assay (Sunday through Monday morning) for wave 2, a Wednesday fasting urine assay for wave 3 and a Friday fasting urine assay for wave 4. Urinary β_2_-MG and MA excretion were measured in waves 1–4. An elevated β_2_-MG > 0.2 mg/L was used to define renal tubular impairment [28] and an elevated MA ≥ 20 mg/L was used to define renal glomerular impairment [29].

### 2.3. Assessment of Adherence to the Mediterranean Diet

The 16-item Mediterranean Diet Quality Index in children and adolescents (KIDMED) [13] was used to assess Mediterranean diet adherence among children aged 6–8 years at baseline. A total of 12 items with positive implications for the Mediterranean diet were designated with a +1 value, while 4 items with a negative meaning were designated with a −1 value. The summed index ranged from 0 to 12 points and can be classified into 3 grades: (1) 0–3, low Mediterranean diet adherence; (2) 4–7, intermediate Mediterranean diet adherence (needs to be improved); (3) 8–12, optimal/good Mediterranean diet adherence.

### 2.4. Data Collection of Covariates

Anthropometric measurements including weight, height, body mass index (BMI), systolic blood pressure (SBP) and diastolic blood pressure (DBP) were analyzed in this study. Height z-scores, weight z-scores and BMI z-scores were calculated using 2007 WHO criteria, and age- and sex-specific SBP z-scores were determined. Lifestyle covariates included sleep duration, screen time and physical activity by parental self-administrated questionnaires (Children’s Sleep Habits Questionnaire [CSHQ] [30,31]; the Chinese version of the Children’s Leisure Activities Study Survey [CLASS-C] [25,32]). A sleep duration of <10 h/d was used to define short sleep. Computer/cell-phone screen time of ≥2 h/d was used to define long screen time. Physical activity of <1 h/d was used to define insufficient physical activity [25].

### 2.5. Statistical Analysis

**Figure 1:**
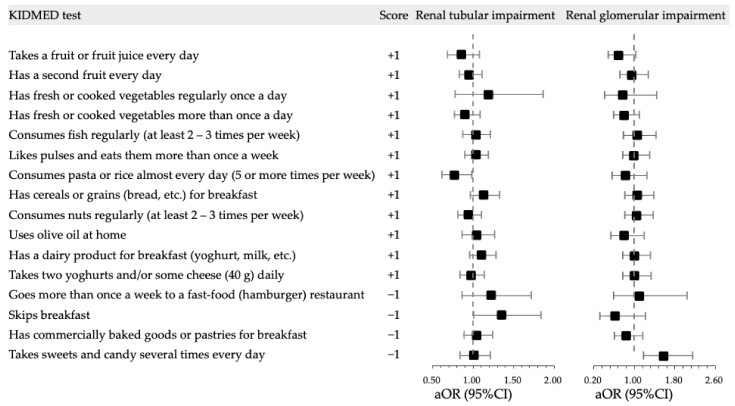
Forest plot for longitudinal associations of 16 items in KIDMED test with transient kidney impairment. All 16 items of the KIDMED test were included in the multivariable models, adjusting for sex, age, body mass index z-score, systolic blood pressure z-score, screen time, sleep duration and physical activity level, while the weekday of the urine assay was included as a random effect.

The main outcomes were the repeated measurements of urinary β_2_-MG and MA excretion and the categorical transient renal tubular and glomerular impairment. Descriptive statistics are presented according to the KIDMED index (Mediterranean diet adherence). Variables with missing values were handled using multiple imputations, and a total of 50 complete datasets were generated for the final analysis. Sex or lifestyle covariates are presented as counts and percentages. Z-scores of height, weight and BMI, SBP and DBP are described as mean ± standard deviation (SD), and urinary β_2_-MG and MA excretion are described as median and interquartile range (IQR). The *χ*^2^ test, analysis of variance and Kruskal–Wallis test were performed to compare the differences between the three groups of poor, intermediate, and good adherence to the Mediterranean diet. We generated linear mixed-effects models to determine the associations between renal impairment indicators and KIDMED scores with estimated coefficients and 95% confidence intervals (95%CI). The longitudinal associations of kidney impairment and KIDMED index were determined using generalized linear mixed-effects models with crude and adjusted odds ratios (cOR and aOR) and 95%CIs. Multivariable models were adjusted for sex, age, BMI z-score, SBP z-score, screen time, sleep duration and physical activity level, while the weekday of the urine assay was included as a random effect. The results in Table 1 are based on the first imputed dataset, while Table 2 and Table 3 and Figure 1 contain valid statistical inferences of the parameters based on 50 datasets using PROC MIANALYZE, and Table 4 is also based on the first imputed dataset. Statistical significance was determined using a two-tailed *p* value of 0.05. All analyses were performed using Statistical Analysis System V.9.4 (SAS Institute Inc., Cary, NC, USA).

## 3. Results

### 3.1. Sociodemographic Characteristics

This study enrolled 1914 children of 6.6 ± 0.3 years old with 7 ± 2 scores of KIDMED test at baseline. Poor, intermediate, and good adherence rates for the Mediterranean diet were 9.0% (KIDMED index 0–3), 54.4% (KIDMED index 4–7) and 36.5% (KIDMED index 8–12), respectively. A short sleep duration was more prevalent in children with lower Mediterranean diet adherence, while there were no significant differences in sex, age, height z-score, weight z-score, BMI z-score, SBP, DBP, long screen time or insufficient physical activity between the three groups. We observed significant differences between the three groups in wave 3 of the urinary β_2_-MG excretion, and in waves 1–2 of the urinary MA excretion between the three groups (Table 1). The prevalence of transient renal tubular impairment (elevated β_2_-MG > 0.2 mg/L) was 8.8% (168 of 1914), 15.9% (277 of 1746), 25.7% (425 of 1651) and 29.0% (481 of 1657) from wave 1 to 4, while that of transient renal glomerular impairment (elevated MA ≥ 20 mg/L) was 5.6% (107 of 1914), 5.5% (96 of 1746), 4.4% (72 of 1651) and 4.1% (68 of 1657), respectively.

### 3.2. Linear Associations of Kidney Impairment Indicators with KIDMED Scores

Linear mixed-effects models were generated to determine associations between the indicators of kidney impairment and the KIDMED scores. The results in the unadjusted model 1 and the adjusted model 2 (adjusting for sex, age, and BMI z-score) showed that the urinary MA excretion was associated with the KIDMED score, whereas the urinary β_2_-MG excretion was not significantly associated with the KIDMED score. The adjusted model 3 showed that a higher urinary MA excretion was inversely associated with a higher KIDMED score (β = −0.216, 95%CI: −0.358, −0.074, *p* = 0.003), adjusting for sex, age, BMI z-score, SBP z-score, screen time, sleep duration and physical activity (Table 2).

### 3.3. Longitudinal Associations of Kidney Impairment with KIDMED Index

The results of the generalized linear mixed-effects models were consistent with the linear models. As the KIDMED index increased (with better Mediterranean diet adherence), the children were less likely to develop renal glomerular impairment. For renal tubular impairment, we did not observe a significant longitudinal association with the KIDMED index. In the unadjusted model 1 and the adjusted model 2 (adjusting for sex, age and BMI z-score), taking the KIDMED index 0–3 as a reference, the effect of the KIDMED index 4–7 on renal glomerular impairment showed marginal significance, while the effect of the KIDMED index 8–12 showed consistent significance. The results from model 3 showed the likelihood of renal glomerular impairment among children with intermediate Mediterranean diet adherence (aOR = 0.68, 95%CI: 0.47, 0.99, *p* = 0.044) and those with good Mediterranean diet adherence (aOR = 0.60, 95%CI: 0.40, 0.90, *p* = 0.014), adjusting for sex, age, BMI z-score, SBP z-score, screen time, sleep duration, and physical activity (Table 3).

### 3.4. Longitudinal Associations of Kidney Impairment with KIDMED Test

We further presented the frequency of children with kidney impairment in terms of each item of the KIDMED test (Table 4) and the association of kidney impairment with each item of KIDMED test (Figure 1). Adjusting for sex, age, BMI z-score, SBP z-score, screen time, sleep duration, and physical activity, renal tubular impairment was less likely to occur among those that consumed pasta or rice almost every day (aOR = 0.77, 95%CI: 0.61, 0.98, *p* = 0.033) and more likely to occur among those that skipped breakfast (aOR = 1.35, 95%CI: 1.00, 1.83, *p* = 0.050). Children who took sweets and candy several times every day had a greater risk to develop renal glomerular impairment (aOR = 1.59, 95%CI: 1.18, 2.15, *p* = 0.003) (Figure 1).

## 4. Discussion

The study used longitudinal data from the general pediatric population in China with a considerably large sample size to determine the associations between Mediterranean diet adherence (assessed using the KIDMED index) and transient kidney impairment. We found that children with a higher KIDMED index were less likely to develop renal glomerular impairment. Good adherence to the Mediterranean diet is associated with a 40% lower risk of having renal glomerular impairment, after adjusting for demographic and lifestyle covariates, including sex, age, BMI z-score, SBP z-score, screen time, sleep duration and physical activity. This finding underscores a major need for early development and adherence to a healthy eating pattern to enhance kidney health in children.

Poor, intermediate, and good adherence rates for the Mediterranean diet were 9.0% (KIDMED index 0–3), 54.4% (KIDMED index 4–7) and 36.5% (KIDMED index 8–12), respectively, while the proportions in the KIDMED-developed population of Spanish children aged 2–14 years were 2.9%, 48.6% and 48.5% [13]. The rate differences suggest slightly lower Mediterranean diet adherence compared with Spanish children. Studies on the assessment of Mediterranean diet adherence have mainly been conducted among Mediterranean European countries such as Spain, Greece, Chile, Portugal, Lebanon and Italy with mean KIDMED scores ranging from 5 to 8 [8], which is similar to our mean KIDMED scores of 7. These results demonstrate the good applicability of the KIDMED in the Chinese pediatric population. Moreover, we observed that a short sleep duration was more prevalent in the context of lower Mediterranean diet adherence. A cross-sectional study among 503 university students reported that good Mediterranean diet adherence was associated with overall good sleep quality and sleep composition, which was associated with the intake of foods rich in melatonin, tryptophan and phytonutrients [33].

For the linear association of the indicators of kidney impairment and the Mediterranean diet adherence, we observed a consistent longitudinal negative association between the KIDMED score and urinary MA excretion in the unadjusted analyses, with respect to the adjusted sex, age, BMI z-score, SBP z-score, screen time, sleep duration and physical activity. This result is consistent with a cross-sectional study of Greek adolescents that reported an inverse correlation between the KIDMED score and albumin to creatinine ratio (ACR, r = −0.111, *p* = 0.041) [11]. The kidney contributes to many physiological and biological mechanisms in the body [34], while microalbuminuria (renal glomerular impairment) is associated with systemic inflammation of the body and endothelial dysfunction [35]. Early damage to the glomerular filtration barrier results in impaired size and charge selectivity, leading to increased MA excretion [36]. One pathophysiological explanation for this negative correlation is that the Mediterranean diet has the endothelial protective and anti-atherosclerotic properties [11,37]. No significant longitudinal associations between the KIDMED score and urinary β_2_-MG excretion were observed. The difference in the molecular mass of β_2_-MG (11.8 kDa) and MA (67 kDa) partly explains the differences associated with the Mediterranean diet, i.e., the excretion of MA requires more extensive impairment [38]. Furthermore, studies have shown that increased glomerular albumin leakage stimulates proinflammatory and profibrotic signals that directly lead to tubulointerstitial impairment [36]. The exposure of renal tubules to albumin triggers inflammatory responses and toxic effects, leading to interstitial impairment, fibrosis and dysfunction, ultimately resulting in irreversible kidney impairment [36,39]. Therefore, Mediterranean diet adherence may play a broader and more important role in preventing kidney damage.

We further assessed the longitudinal association between transient kidney impairment and Mediterranean diet adherence and validated the stability of the inverse association between renal glomerular impairment and Mediterranean diet adherence. We found that children with moderate or good Mediterranean diet adherence had a 32% and 40% lower risk of glomerular impairment, respectively, compared with children with low Mediterranean diet adherence, adjusting for sex, age, BMI z-score, SBP z-score, screen time, sleep duration and physical activity level. This result provides quantitative evidence for evaluating the direct effect of the Mediterranean diet on glomerular impairment in children. We found a similar non-significant negative association between renal tubular impairment and Mediterranean diet adherence. A further breakdown of the 16-item KIDMED test analysis revealed that eating pasta or rice almost every day was associated with a 23% lower risk of renal tubular impairment. China is a country with a traditional grain-based diet, and most children eat grains more than once a day [4,12], which may be related to the protection of the renal tubular system. We also identified two unhealthy eating habits as leading causes of transient kidney impairment, namely, skipping breakfast increases the risk of tubular impairment by 35% and eating sweets and candies several times a day increases glomerular impairment by 59%. While we did not expect children in Beijing or in China to have absolute adherence to the Mediterranean diet, the KIDMED test involving many healthy compositions are similar to the traditional Chinese dietary pattern or other healthy diet patterns. In this perspective, using KIDMED advocates a healthy dietary pattern rather than promoting a Mediterranean diet per se. The early development of healthy eating patterns and the correction of unhealthy eating habits will help protect kidney health in the general pediatric population with respect to healthy lifestyles.

The major strength of this study was that we used the quantified tool of the KIDMED index to assess Mediterranean diet adherence, thus ensuring the stability of the results. However, our findings are based on the original version of the KIDMED questionnaire [13], and there was a revision in 2019 to emphasize the benefits of consuming whole fruit rather than fruit juice and whole grains [40]. We used appropriate statistical methods such as imputation methods [41] for missing data, linear mixed-effects models for linear associations and generalized linear mixed-effects models for longitudinal associations, to maximize longitudinal information and avoid reporting bias. In addition, we selected and adjusted for key covariates such as sex, age, BMI, SBP and lifestyle factors to assess the effect of the Mediterranean diet on transient kidney impairment. This study was limited by not considering the potential interaction of the two outcomes and other factors of renal function. Urine β_2_-MG and MA excretion were tested by different machines across the study waves, but longitudinal data minimized these effects.

## 5. Conclusions

We present the longitudinal associations of transient kidney impairment with Mediterranean diet adherence in the general Chinese pediatric population. We found that children who adhered to a Mediterranean diet were associated with a reduced risk of transient renal glomerular impairment. Our findings underscore the necessity of the early development of healthy diet patterns and the correction of unhealthy eating habits in children to protect kidney health in the context of more relevant healthy lifestyles.

## Figures and Tables

**Table 1:** Participants’ characteristics categorized by KIDMED index of children aged 6–9 in Beijing, China (N = 1914).

Characteristics	KIDMED Index (Mediterranean Diet Adherence)			*p*
	0–3 (Low *n* = 173)	4–7 (Intermediate *n* = 1042)	8–12 (Good *n* = 699)	
At baseline				
Boy (*n* (%))^1^	90 (52.0)	506 (48.6)	360 (51.5)	0.41
Age (year) ^2^	6.6 ± 0.3	6.6 ± 0.3	6.6 ± 0.3	0.64
Height z-score ^2^	0.73 ± 0.94	0.62 ± 0.96	0.71 ± 0.95	0.12
Weight z-score ^2^	0.78 ± 1.42	0.67 ± 1.43	0.74 ± 1.38	0.41
Body mass index z-score ^2^	0.45 ± 1.58	0.37 ± 1.54	0.41 ± 1.52	0.76
Systolic blood pressure (mmHg) ^2^	100 ± 9	101 ± 9	101 ± 8	0.27
Diastolic blood pressure (mmHg) ^2^	56 ± 6	56 ± 6	56 ± 6	0.60
Short sleep (<10 h/d) ^1^	149 (86.1)	792 (76.0)	500 (71.5)	<0.001
Long screen time (≥2 h/d) ^1^	10 (5.8)	55 (5.3)	30 (4.3)	0.57
Insufficient physical activity (<1 h/d) ^1^	136 (78.6)	789 (75.7)	526 (75.3)	0.65
Urinary β_2_-MG excretion (mg/L) ^3^				
Wave 1	0.09 (0.04–0.15)	0.08 (0.04–0.13)	0.08 (0.04–0.12)	0.34
Wave 2	0.15 (0.12–0.18)	0.14 (0.12–0.18)	0.14 (0.12–0.18)	0.15
Wave 3	0.16 (0.13–0.21)	0.17 (0.13–0.21)	0.16 (0.13–0.20)	0.017
Wave 4	0.17 (0.13–0.22)	0.16 (0.13–0.21)	0.16 (0.13–0.21)	0.42
Urinary MA excretion (mg/L) ^3^				
Wave 1	9.67 (6.57–13.14)	9.19 (6.54–12.78)	8.76 (6.30–11.80)	0.027
Wave 2	7.15 (6.20–9.20)	6.70 (6.10–8.50)	6.60 (6.00–8.40)	0.046
Wave 3	7.30 (6.30–9.20)	7.10 (6.30–9.50)	7.00 (6.20–8.80)	0.10
Wave 4	6.90 (6.00–10.50)	6.80 (6.00–9.00)	6.65 (6.00–8.70)	0.089

KIDMED: the 16-item Mediterranean Diet Quality Index in children and adolescents; β_2_-MG: β_2_-microglobulin; MA: microalbumin.^1^ Comparison by KIDMED group using χ^2^ test. ^2^ Means and standard deviations (SDs) compared between KIDMED groups using analysis of variance. ^3^ Medians and interquartile ranges (IQRs) compared between KIDMED groups using Kruskal–Wallis test.

**Table 2:** Linear associations of indicators of kidney impairment with KIDMED scores among children aged 6–9 in Beijing, China using linear mixed-effects models.

Dependent Variable	Independent Variable	Model 1		Model 2		Model 3	
		Estimate (95%CI)	*p*	Estimate (95%CI)	*p*	Estimate (95%CI)	*p*
**Urinary β_2_-MG excretion**	KIDMED score	−0.001 (−0.003, 0.001)	0.20	−0.001 (−0.002, 0.001)	0.28	−0.001 (−0.003, 0)	0.13
**Urinary MA excretion**	KIDMED score	−0.218 (−0.359, −0.076)	0.003	−0.206 (−0.347, −0.065)	0.004	−0.216 (−0.358, −0.074)	0.003

Model 1: unadjusted; model 2: adjusted for sex, age, body mass index z-score; model 3: based on model 2, further adjusted for systolic blood pressure z-score, screen time, sleep duration and physical activity level. (KIDMED: 16-item Mediterranean Diet Quality Index in children and adolescents; β_2_-MG: β_2_-microglobulin; MA: microalbumin; CI: confidence interval. All models included one random effect: the weekday of the urine assay).

**Table 3:** Longitudinal associations of kidney impairment with KIDMED index among children aged 6–9 in Beijing, China using generalized linear mixed-effects models.

Dependent Variable	Independent Variable	Model 1		Model 2		Model 3	
		cOR (95%CI)	*p*	aOR (95%CI)	*p*	aOR (95%CI)	*p*
**Renal tubular impairment**	KIDMED index 0–3	1		1		1	
	KIDMED index 4–7	0.90 (0.72, 1.12)	0.33	0.90 (0.72, 1.12)	0.35	0.88 (0.70, 1.10)	0.25
	KIDMED index 8–12	0.86 (0.69, 1.09)	0.22	0.87 (0.69, 1.10)	0.26	0.84 (0.67, 1.07)	0.16
**Renal glomerular impairment**	KIDMED index 0–3	1		1		1	
	KIDMED index 4–7	0.71 (0.50, 1.03)	0.068	0.70 (0.48, 1.00)	0.051	0.68 (0.47, 0.99)	0.044
	KIDMED index 8–12	0.61 (0.41, 0.91)	0.016	0.62 (0.41, 0.92)	0.018	0.60 (0.40, 0.90)	0.014

Model 1: unadjusted; model 2: adjusted for sex, age, body mass index z-score; model 3: based on model 2, further adjusting for+ systolic blood pressure z-score, screen time, sleep duration and physical activity level. (KIDMED: 16-item Mediterranean Diet Quality Index in children and adolescents; cOR: crude odds ratio; aOR: adjusted odds ratio; CI: confidence interval. All models included one random effect: the weekday of the urine assay).

**Table 4:** Frequency of item-specific KIDMED test with kidney impairment (N = 6968).

KIDMED Test Item	Score	Renal Tubular Impairment *n* (%)	Renal Glomerular Impairment *n* (%)
Takes a fruit or fruit juice every day			
Yes	+1	1190 (19.1)	294 (4.7)
No	0	161 (21.8)	49 (6.6)
Has a second fruit every day			
Yes	+1	752 (18.7)	187 (4.6)
No	0	599 (20.4)	156 (5.3)
Has fresh or cooked vegetables regularly once a day			
Yes	+1	1312 (19.4)	327 (4.8)
No	0	39 (18.0)	16 (7.4)
Has fresh or cooked vegetables more than once a day			
Yes	+1	1044 (19.0)	244 (4.4)
No	0	307 (20.8)	99 (6.7)
Consumes fish regularly (at least 2–3 times per week)			
Yes	+1	409 (19.5)	99 (4.7)
No	0	942 (19.4)	244 (5.0)
Likes pulses and eats them more than once a week			
Yes	+1	813 (19.4)	200 (4.8)
No	0	538 (19.4)	143 (5.2)
Consumes pasta or rice almost every day (5 or more times per week)			
Yes	+1	1210 (18.9)	308 (4.8)
No	0	141 (24.5)	35 (6.1)
Has cereals or grains (bread, etc.) for breakfast			
Yes	+1	851 (20.1)	208 (4.9)
No	0	500 (18.3)	135 (4.9)
Consumes nuts regularly (at least 2–3 times per week)			
Yes	+1	516 (19.4)	128 (4.8)
No	0	835 (19.4)	215 (5.0)
Uses olive oil at home			
Yes	+1	196 (19.0)	47 (4.6)
No	0	1155 (19.5)	296 (5.0)
Has a dairy product for breakfast (yoghurt, milk, etc.)			
Yes	+1	872 (19.8)	202 (4.6)
No	0	479 (18.7)	141 (5.5)
Takes two yoghurts and/or some cheese (40 g) daily			
Yes	+1	464 (19.4)	104 (4.4)
No	0	887 (19.4)	239 (5.2)
Goes more than once a week to a fast-food (hamburger) restaurant			
Yes	−1	54 (23.0)	14 (6.0)
No	0	1297 (19.3)	329 (4.9)
Skips breakfast			
Yes	−1	78 (22.8)	12 (3.5)
No	0	1273 (19.2)	331 (5.0)
Has commercially baked goods or pastries for breakfast			
Yes	−1	322 (19.7)	76 (4.6)
No	0	1029 (19.3)	267 (5.0)
Takes sweets and candy several times every day			
Yes	−1	199 (19.7)	76 (7.5)
No	0	1152 (19.3)	267 (4.5)

KIDMED: the 16-item Mediterranean Diet Quality Index in children and adolescents.

## Data Availability

The data that support the findings of this study are not publicly available but are available from the corresponding author on reasonable request.
